# Use of Binary Cumulative Sums and Moving Averages in Nosocomial Infection Cluster Detection^1^

**DOI:** 10.3201/eid0812.010514

**Published:** 2002-12

**Authors:** Samuel M. Brown, James C. Benneyan, Daniel A. Theobald, Kenneth Sands, Matthew T. Hahn, Gail A. Potter-Bynoe, John M. Stelling, Thomas F. O'Brien, Donald A. Goldmann

**Affiliations:** * Massachusetts General Hospital, Boston, Massachusetts, USA; † Northeastern University, Boston, Massachusetts, USA; ‡ Vecna Technologies, Inc., Hyattsville, Maryland, USA; § Beth Israel Deaconess Medical Center, Boston, Massachusetts, USA; ¶ Children's Hospital, Boston, Massachusetts, USA; # WHO Collaborating Center for Antimicrobial Resistance Surveillance, Boston, Massachusetts, USA; ** Brigham and Women's Hospital, Boston, Massachusetts, USA

**Keywords:** Cross-infection, disease surveillance, outbreak detection, cumulative sums, moving averages

## Abstract

Clusters of nosocomial infection often occur undetected, at substantial cost to the medical system and individual patients. We evaluated binary cumulative sum (CUSUM) and moving average (MA) control charts for automated detection of nosocomial clusters. We selected two outbreaks with genotyped strains and used resistance as inputs to the control charts. We identified design parameters for the CUSUM and MA (window size, *k*, α, β, *p_0_, p_1_*) that detected both outbreaks, then calculated an associated positive predictive value (PPV) and time until detection (TUD) for sensitive charts. For CUSUM, optimal performance (high PPV, low TUD, fully sensitive) was for 0.1 <α ≤0.25 and 0.2 <β <0.25, with *p*_0_ = 0.05, with a mean TUD of 20 (range 8–43) isolates. Mean PPV was 96.5% (relaxed criteria) to 82.6% (strict criteria). MAs had a mean PPV of 88.5% (relaxed criteria) to 46.1% (strict criteria). CUSUM and MA may be useful techniques for automated surveillance of resistant infections.

Nosocomial infections afflict 2 to 5 million patients in the United States annually and contribute to approximately 88,000 deaths (1,2). These infections are the second most frequent adverse effect of hospitalization (3,4). In most instances such infections are isolated, though studies have reported that from 2% (5,6) to 20% (7) to 60% (8) occur in clusters. A minimal estimate of the epidemic nosocomial infection burden is thus 40,000 cases annually (2% of 2,000,000), while a maximal estimate is conceivably five times that figure or more.

Most hospitals in the United States will have at least one outbreak per year, and large referral hospitals may have several (9). Nosocomial infection clusters can be difficult to diagnose and detect (5), which can have serious ramifications (10). Although options for computerized surveillance are increasing (11–15), many current methods for outbreak detection are effective only when substantial time has elapsed from the actual events. Techniques are often poorly automated (16–18), and few sophisticated cluster detection techniques have been employed in nosocomial infection surveillance (19–21). 

Cumulative sums (CUSUMs) are statistical tools, based on a type of sequential hypothesis test, that were originally used in manufacturing processes to monitor production defect rates (22–24). Increments are added or decrements are subtracted from a running total over time, according to measurements of quality of serial items. The behavior of this cumulative sum is tracked until one of two conditions is met, with CUSUM values beyond these thresholds signaling either 1) a statistically significant change in quality to some prespecified level or 2) acceptance of the hypothesis of no change. CUSUMs have been used for several decades in health care settings, including for tracking operator improvements in performing procedure (25–27), monitoring fever curves in neutropenic patients (28), and detecting community *Salmonella* outbreaks (15). Several forms exist, including a so-called binary or Bernoulli CUSUM in which failure is rated as 1 and success as 0, a coefficient is subtracted, and the resulting values are added to the CUSUM. This binary form has not to our knowledge been applied to outbreak detection.

Moving averages (MAs) are in wide use in several fields, such as economics, where methods sensitive to sudden changes and filtering out white noise are required. Thus, for instance, economic indicators may be analyzed, with a MA calculated for the most recent values and compared with the historical mean for that indicator. An MA much higher than the historical mean indicates a statistical increase. MAs also are used in manufacturing quality control for the same reason (28). Although various MA techniques have been applied to disease rates in public health surveillance (29), they have not previously been applied to monitor changes in strain characteristics, such as antimicrobial resistance.

We hypothesized that by treating antimicrobial resistance as the quality indicator of individual isolates, these techniques could be used to detect nosocomial clusters. Both techniques have been demonstrated in the quality control literature to be more sensitive to small rate changes than conventional p-type charts (22–24,30). We evaluated the performance of these techniques in simulated real-time detection of two genotypically characterized outbreaks of nosocomial infection caused by antimicrobial-resistant bacteria.

## Methods

### Outbreaks Investigated

The study hospital is a 330-bed tertiary-care pediatric facility in the northeastern United States. We selected all investigated nosocomial outbreaks of antibiotic-resistant bacteria in the study hospital for which genotyping data were available for the period 1995–2000, inclusive. An outbreak with genotyped organisms from 1997 was excluded because the causative agent, *Pseudomonas aeruginosa,* was sensitive to all standard therapeutic agents. This cluster was thus not a candidate for detection with our techniques. A line listing of all patients, with isolates, from both outbreaks is presented in the Table.

**Table:** Cluster patients with isolates, dates, and sensitivities^a^

Patient	Culture date	Body site	PFGE type	Resistance phenotype^b^
MRSA				
O1-1	1/22/99	wd,bl	E	cli ERY tcy van SAM FEP OXA sxt CZO AMC amk
O1-2	7/10/99	no,ax	D	CLI ERY TCY van OXA sxt AMK
O1-3	7/10/99	sp	D	CLI ERY TCY van SAM FEP OXA sxt CZO AMC AMK
O1-4	8/23/99	wd	C	CLI ERY TCY van SAM FEP OXA sxt CZO AMC AMK
O1-5	9/3/99	wd	C	CLI ERY TCY van SAM FEP OXA sxt CZO AMC AMK
O1-6	9/6/99	wd	C	CLI ERY TCY van SAM FEP OXA sxt CZO AMC AMK
O1-7	9/13/99	bl	C	CLI ERY TCY van OXA sxt AMK
VRE				
O2-1	1/20/00	bl	non-B	VAN amc AMP
O2-2	5/12/00	st	B	VAN amc AMP
O2-3	5/14/00	fl	B	VAN amc AMP
O2-4	5/18/00	st	B	VAN amc AMP
O2-5	5/19/00	ti,st	B	VAN amc AMP TCY chl IPM nit
O2-6	5/24/00	st	B	VAN amc AMP
O2-7	6/23/00	fl	non-B	VAN AMC AMP

^a^MRSA, methicillin-resistant *Staphylococcus aureus*; PFGE, pulsed-field gel electrophoresis; O1, outbreak 1; O2, outbreak 2; VRE, vancomycin-resistant enteroccocus; bl, blood; sp, sputum; st, stool; wd, wound; ti, tissue; ax, axilla; no, nose; fl, fluid. ^b^Antibiotic codes in capital letters are resistant results; those in lowercase letters are susceptible: AMC, amoxicillin/clavulanate; AMP, ampicillin; AMK, amikacin; CLI, clindamycin; CZO, cefazolin; ERY, erythromycin; FEP, cefepime; OXA, oxacillin; SAM, ampicillin/sulbactam; SXT, trimethoprim/sulfamethoxazole; TCY, tetracycline; VAN, vancomycin.

The Institutional Review Board of the study hospital authorized us to perform this study without obtaining informed consent. All patient identifiers were either deleted or irreversibly encrypted to ensure confidentiality.

#### Outbreak 1

An outbreak of surgical site infections caused by methicillin-resistant Staphylococcus aureus (MRSA) occurred in August through September 1999 in patients after cardiac surgery. Approximately 800 such surgeries are performed annually in the study hospital. Immediately after surgery, patients are cared for in the cardiovascular intensive care unit (CICU), which has 23 beds, 1,550 admissions per year, and an average length of stay of 4.4 days. After they are stabilized, the patients are transferred to the cardiac surgery ward (28 beds, >2,300 admissions per year; and average length of stay, 3 days). A single genotype of MRSA was isolated from four patients with evidence of deep/organ-space surgical infection after cardiac surgery. One of the genotypically identical isolates (O3-2) was detected by admission screening culture at another hospital to which the patient had been transferred. Another isolate (O3-7) was detected in a blood culture obtained at the hospital to which the patient had been transferred. Two surgical patients without clinical infection were colonized with isolates of a second genotype. Methicillin resistance was defined as a MIC of oxacillin of >0.5 μg/ml. All isolates of Staphylococcus aureus from any body site from the CICU and cardiac surgical ward were included in the analyses.

#### Outbreak 2

An outbreak of vancomycin-resistant enterococcus (VRE) occurred in May through June 2000 involving two units: the bone marrow transplant unit and the general pediatric intensive care unit PICU. The bone marrow transplant unit is a 13-bed unit providing hematopoietic stem-cell transplantation. It has approximately 260 admissions per year, with an average length of stay of 12.9 days. When patients require ICU care, they are transferred to specially ventilated rooms in the PICU. The PICU is an 18-bed multidisciplinary unit, with approximately 1,650 admissions per year, and an average length of stay of 3.2 days. In May 2000, a patient colonized with VRE in the bone marrow transplant unit was transferred to the PICU. Other cases of VRE colonization or infection were detected in both the bone marrow transplant unit (4 cases) and the PICU (3 cases). Isolates of *Enteroccocus faecium* from five patients were demonstrated to be genotypically identical. Vancomycin resistance was defined as a MIC of vancomycin of ≥16 µg/ml. All isolates of *E.faecium* or unspeciated *Enterococcus* from any body site on the affected units were included in the analyses. Genotyping was performed by ARUP Laboratories (Salt Lake City, UT). Genotypic identity was defined according to a published procedure (31).

### Data Acquisition

Records for all inpatient cultures were downloaded from the study hospital’s information system for January 1995–September 2000 into WHONET 5.0 (WHO Collaborating Center, Boston, MA). Species identification had been performed per standard laboratory procedures. Antibiotic sensitivities had been performed by measurement MIC with a MicroScan Walkaway-96 (Dade Behring, Inc., Deerfield, IL). Standard Kirby-Bauer technique was used when an organism failed to grow sufficiently to perform MIC analysis. Only final susceptibility readings were included. Susceptibility cutoffs were defined according to National Committee for Clinical Laboratory Standards (32). Indication for culture was specified as either clinical (C), routine surveillance (R), or outbreak investigation (O). Clinical cultures were ordered by treating physicians for care of the individual patient. Routine surveillance cultures included weekly stool screens for VRE and sentinel event screens. Infection control policy at the study hospital was to screen a high-risk unit (ICU or bone marrow transplant unit) if a patient was found to have new MRSA or VRE colonization or infection. Outbreak investigation cultures were those taken as part of a formal or informal outbreak investigation. Culture indications were determined from infection control records.

### Data Analysis

**Figure 1:**
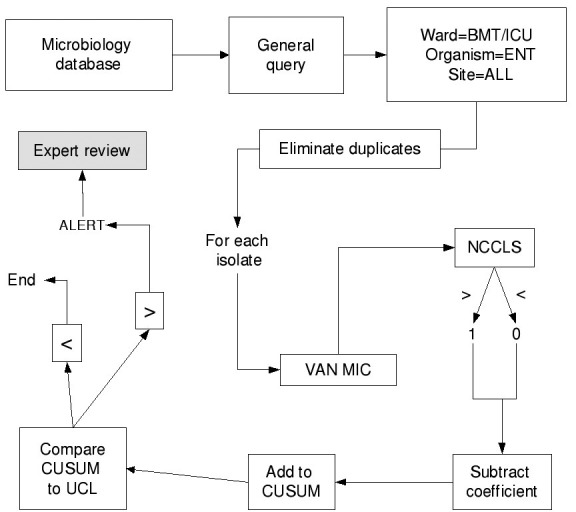
Data processing methodology for cumulative sums. BMT, bone marrow transplant unit; ICU, intensive care unit; ENT, enterococcus; VAN MIC, vancomycin minimum inhibitory concentration; NCCLS, National Committee on Laboratory Standards antibiotic susceptibility breakpoint; CUSUM, cumulative sum; UCL, upper confidence limit.

**Figure 2:**
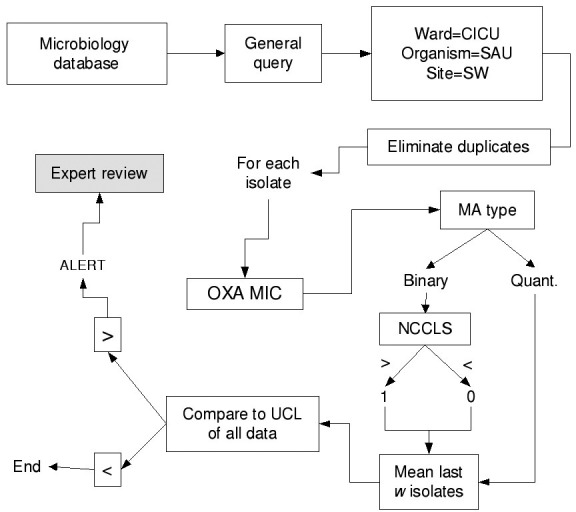
Data-processing methodology for moving averages. CICU, cardiac intensive care unit; SAU, *S. aureus*; SW, surgical wound; OXA MIC, oxacillin minimum inhibitory concentration; MA, moving average chart; NCCLS, National Committee on Laboratory Standards, antibiotic susceptibility breakpoint; UCL, upper control limit.

 Isolates of the same species from a given patient within 60 days of the previous isolate were excluded as duplicate isolates. All isolates of *E. faecium*, enterococcus, and *S. aureus* from the affected units were parsed by the BugCruncher program (Vecna Technologies, Hyattsville, MD) in the manner depicted in Figures 1 and 2. The resistance value (for binary tests 0 = susceptible or 1 = nonsusceptible; for quantitative tests, the actual MIC) for each isolate was then passed to CUSUM (binary only) or MA (binary and quantitative) modules, where alerts were generated on the basis of control limits. Test statistics and control limits were recalculated with the addition of each new isolate and processed in chronological order.

Each type of chart is calculated based on several design parameters (*w* and *k* for MA; α, β, *p*_0_, *p*_1_ for CUSUM). To explore performance robustness under various conditions, we selected a reasonable range of values for the control parameters for CUSUM (0.01 ≤α ≤0.25; 0.01 ≤β ≤.25; 0.01 ≤*p*_0_ ≤ 0.25; 0.01 ≤*p*_1_ ≤0.25) and MA (5 ≤*w* ≤90 and 1 ≤*k* ≤4) charts. Positive predictive value (PPV) was calculated for those design parameter values that detected both outbreaks. Further detail on these statistical methods and the formulae used for calculating their test statistics and detection thresholds are presented in the Appendix .

 To validate the empirically derived design parameters in terms of theoretic performance, we then calculated the out-of-control (an actual change in incidence) and in-control (no change in incidence) time until detection (TUD) for the sets of design parameters that detected both outbreaks. We used standard methods for calculating TUDs, employing a Monte Carlo simulation program we wrote for that purpose. Simulations were run over 10,000 iterations.

Two definitions of cluster detection were used: generation of an alert at the second outbreak isolate (isolate-level detection) or during the first month of the outbreak (month-level detection). Positive predictive value (percent of detected events considered relevant) was calculated in the following manner (33) all detected events previously unnoted by infection control personnel were evaluated independently by two hospital epidemiologists (KS, DG). The epidemiologists classified each event as A) initiate investigation, B) monitor situation, or C) ignore. A “C” rating from both epidemiologists or a “B” from one and a “C” from the other was considered a false-positive result. True positives were divided into positives by strict criteria (receiving an “A” rating) and by relaxed criteria (receiving at least “B” ratings from both epidemiologists). PPVs were calculated by strict and relaxed criteria separately.

## Results

### Cluster Descriptions

The dataset contained a total of 6,382 positive cultures of any organism (from 3,346 different patients) from the units affected by the outbreak of oxacillin-resistant S. aureus. Of those, 728 (from 323 patients) were S. aureus. Of the 323 unique isolates of S. aureus in the affected units, 14 (4.3%) were oxacillin resistant, whereas for the hospital as a whole 84 (4.2%) of 1,983 S. aureus isolates were oxacillin resistant.

The dataset contained a total of 9,012 positive cultures of any organism (from 4,315 patients) from the units affected by the outbreak of vancomycin-resistant enterococcus. In the affected units, 21 (14.1%) of 149 enterococcal isolates were vancomycin resistant, whereas for the entire hospital 41 (5.3%) of 768 enterococcal isolates were vancomycin resistant.

For all implicated units, the 15 most common bacterial species represented 4,948 unique isolates, an average of 18 per unit per month. Overall 165 different organisms were isolated, 74 of them representing only three or fewer isolates over the 69 months included in the dataset.

### CUSUMs

**Figure 3:**
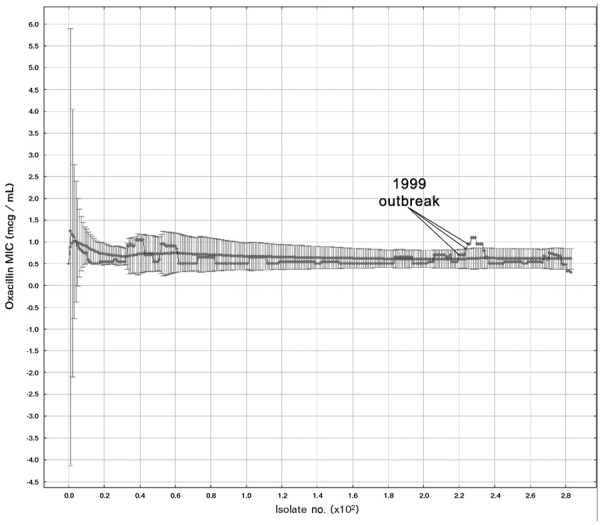
Cumulative sum test iteration detecting an outbreak of vancomycin-resistant enterococcus. Test parameters were *p*_0_ = 0.05, p_1_ = 0.15, α = 0.15, β = 0.2, and included enterococcal isolates from all body sites from the affected wards, excluding strains found during outbreak investigation.

Several CUSUM charts proved capable of detecting both outbreaks by the second isolate. Figure 3 displays a representative CUSUM chart, which detected the VRE outbreak early in its course. Maximal performance robustness was obtained when 0.1<α<2 and 0.2<β<0.25, with *p*_0_ = 0.05. Values of β<0.2 were associated with poor performance.

Monte Carlo simulations, run with *p*_1_ = 0.2 over the sets of design parameters that performed most robustly, yielded an out-of-control TUD ranging from 8 to 45 isolates (average 20.4), and an in-control TUD, ranging from 55 to 2,390 isolates (average 427). Both the out-of-control TUD and in-control TUD decreased with higher values of α; for α = 0.2 or 0.25, the in-control TUD ranged from 55 to 88; whereas at α = 0.1, it ranged from 184 to 306 isolates.

The mean PPV of CUSUM techniques ranged from 96.5% (relaxed criteria) to 82.6% (strict criteria). Lower values for α were associated with higher PPV. On average, the sensitive control charts generated 9.5 novel alerts over the 69 months of the study period, or 1.6 events per year for all involved units and organisms (enterococcus, *S. aureus*).

### Moving Averages

For MA control charts, only those which used quantitative MICs (vancomycin: 2–16 μg/mL; oxacillin: 0.25–4 μg/mL) were capable of detecting both outbreaks; no binary (susceptible = 0; nonsusceptible = 1) MA charts detected both outbreaks. Sensitive window sizes (w, the number of isolates considered in calculating the MA) varied from 5 to 30 isolates. Parameter sets with larger window sizes failed to detect both outbreaks.

Monte Carlo simulations for the design parameters that detected both outbreaks, assuming a change in MICs of one standard deviation, yielded an out-of-control TUD ranging from 4 to 10,796 isolates (mean 1,568; median 14), and an in-control TUD ranging from 11 to 25,488 (mean 4,006; median 180). For *k* < 4, the mean out-of-control TUD was 14, while the mean in-control TUD was 350 isolates.

**Figure 4:**
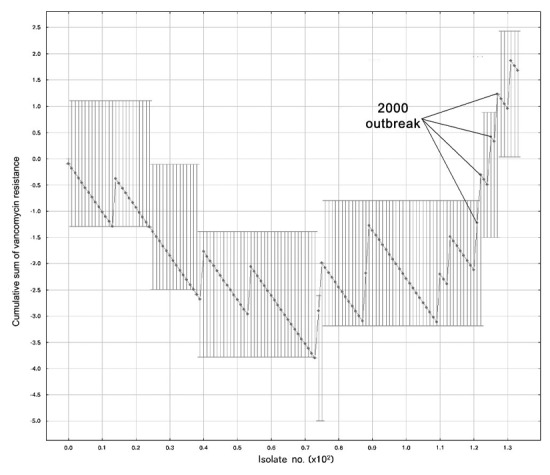
Moving average test iteration detecting an outbreak of methicillin-resistant *Staphylococcus aureus*. Test parameters were *w* = 10, *k* = 4, and included all *S. aureus* from all body sites from the affected wards, excluding strains found during outbreak investigations. MIC, minimum inhibitory concentration

Figure 4 displays a representative MA test combination that detected the MRSA outbreak by the second isolate. The mean PPV ranged from 88.5% (relaxed criteria) to 46.1% (strict criteria). On average, sensitive MA charts generated 10.9 novel alerts over the entire study period, or 1.9 per year for all units and organisms studied.

## Discussion

 We illustrated the performance of a system designed for real-time monitoring of clinical microbiology data from the hospital laboratory information system. Two techniques borrowed from other domains were capable of detecting two carefully characterized outbreaks in simulated real time. The binary CUSUM proved more robust than MAs.

Many metrics for outbreak detection are based on month of outbreak (11,14,15,17,18,34), whereas in nosocomial outbreaks greater attention to individual cases is probably warranted given the smaller numbers of patients involved, the possibility of early definitive intervention, and the comorbidities of infected patients. The techniques used in this study proved capable of detecting an outbreak before the end of a monthly surveillance period.

 The reproducibility of these findings is of key importance. We used an a priori reasonable set of possible design parameter values, then combined empirical evaluation of their performance with theoretical evaluation via Monte Carlo simulations.

We used only two outbreaks for evaluation, given the difficulty of generating and validating such datasets. A study that investigates larger numbers of similar outbreaks would improve generalizability. The theoretical simulations tend to support the generalizability of the test statistics used, as the empirically robust design parameters were associated with low out-of-control and high in-control TUD values.

The techniques appear most useful when the baseline incidence is relatively low, and it is unclear whether these methods would be applicable in settings where antibiotic-resistant bacteria are more common, as the study hospital had relatively low rates of MRSA and VRE.

The surveillance methods evaluated here are primarily useful for detecting outbreaks caused by resistant organisms. In their current implementation, they would not be useful for settings where outbreaks are caused by organisms whose antibiotic susceptibilities are indistinguishable from those of endemic flora, as in the cluster of *Pseudomonas* excluded from the present study. Additional research would be required to make these methods applicable in those settings.

From a practical perspective, the CUSUM charts detected the outbreaks by the second isolate, a finding corroborated by results of the Monte Carlo simulations. An increased incidence from .05 to .20 would be detected on average within 1.5 actual outbreak isolates for an out-of-control TUD of 10 (best-performing CUSUM), or at the third outbreak isolate for an out-of-control TUD of 20 (mean CUSUM performance). These results, supported empirically and theoretically, are consistent with the goals of nosocomial outbreak detection.

In terms of resources potentially wasted on false-positive results, the CUSUM charts that detected both outbreaks were remarkably accurate, with an average PPV of >80%, even by strict criteria, whereas the MIC MA parameter sets had lower PPVs. According to our calculated PPV for CUSUM, only 1 in 20 alerts would be deemed retrospectively as unworthy of any further evaluation, while 1 in 5 would not be deemed worthy of actual investigation. Assuming an annual rate of 1 alert per organism and unit, 4 units under surveillance, and 15 organisms under surveillance, 60 alerts would be generated annually, of which 12 would not be deemed worthy of attention, approximately one false alarm per month. Slightly more than twice as many would be considered spurious in retrospect on the basis of the MA results.

Using the in-control TUD values to estimate the frequency of spurious results yields a better estimate. With 18 isolates of the 15 most commonly isolated bacteria per unit per month, 4 units under surveillance, we would anticipate 72 isolates per month. The mean in-control TUD value for CUSUM charts is 427, suggesting a false-positive alert once every 5 months, though false-positive alerts are associated with a higher out-of-control TUD. Taking the chart with the lowest out-of-control TUD, the in-control TUD is 55, suggesting a false-positive result slightly more than once per month, similar to our observed rate.

Strengths of this study include the availability of genotyping data for outbreak characterization and the availability of quantitative MICs, the use of practical outcome measures, and combination of empirical and theoretical methods for evaluating test statistics.

An additional problem in validating detection techniques is the lack of a gold standard for determining the relevance of a computer-detected cluster. We chose a practical approach, given the ultimate clinical application of such a system. We may have overestimated the positive predictive value, although we evaluated by both strict and relaxed criteria. At the time of evaluation, reviewers were unaware of events that followed, decreasing the probability of outcome-based bias. A prospective trial of these techniques, with collection of genotyping information, should help to resolve this problem.

Areas for additional research include methods for analyzing duplicate isolates from a single patient, more sophisticated techniques for modeling patient location, accounting robustly for changes in sampling intensity, methods for using quantitative CUSUMs, and the potential need for corrections for interdependence.

CUSUM and MA analyses of antimicrobial resistance proved capable of detecting two important nosocomial outbreaks early in their course in simulated real time. Both methods had relatively high positive predictive values; CUSUM performed better than MA. These analytical techniques may be of value in automated detection of nosocomial outbreaks and should be evaluated in real-time clinical practice.

## Supplementary Material

Appendix Calculating Test Statistics
