# Supplementary material for: Use of Binary Cumulative Sums and Moving Averages in Nosocomial Infection Cluster Detection
Source: Emerg Infect Dis. 2002 Dec;8(12):1426–32. doi: 10.3201/eid0812.010514 (PMC2737829; doi:10.3201/eid0812.010514)
Supplement: Appendix — Calculating Test Statistics [file 01-0514-app-s1.pdf]

Publisher: CDC; Journal: Emerging Infectious Diseases
Article Type: Research; Volume: 8; Issue: 12; Year: 2002; Article ID: 01-0514
DOI: 10.3201/eid0812.010514; TOC Head: Research

Appendix: Calculating Test Statistics

Binary cumulative sum charts, based on the theory of sequential probability ratio tests, monitor a cumulative term that is incremented or decremented by certain amounts for each positive or negative result, respectively, in order to sequentially test between user-specified acceptable and unacceptable rates (35,36) (Equation 1). In our application, the CUSUM statistic S_i is reduced at the time of each isolate by an amount D, a calculated value that depends on the shift we wish to detect, and then increased by 1 for those isolates that are antibiotic resistant. The plotted statistic for the ith isolate, S_i, and the control limit factors h_0 and h_1 are calculated as

$$S_i = \begin{Bmatrix} S_{i-1} - D, & \text{if } X_i = 0 \\ S_{i-1} + 1 - D, & \text{if } X_i = 1 \end{Bmatrix} = S_{i-1} + X_i - D, \qquad (1)$$

$$h_0 = \frac{\ln\left(\frac{1-\alpha}{\beta}\right)}{\ln\left(\frac{p_1}{p_0}\cdot\frac{1-p_0}{1-p_1}\right)}, \text{ and} \qquad (2)$$

$$h_1 = \frac{\ln\left(\frac{1-\beta}{\alpha}\right)}{\ln\left(\frac{p_1}{p_0}\cdot\frac{1-p_0}{1-p_1}\right)}, \qquad 3)$$

where $X_i = 1$ if the ith isolate is resistant and 0 if it is not, the decrement D is computed as

$$D = \frac{\ln\left(\frac{1-p_0}{1-p_1}\right)}{\ln\left(\frac{1-p_0}{p_0}\cdot\frac{p_1}{1-p_1}\right)},$$

α is the desired type I error rate, β is the desired type II error rate, p_0 is the acceptable occurrence rate, p_1 is the unacceptable occurrence rate that is desired to be detected, and $S_0 = 0$ as a starting value.

The cumulative sum then is compared to nonconstant control limits that periodically are recalculated by subtracting h_0 from and adding h_1 to any S_i value that falls outside either limit, resulting in new limits until the next such violation and starting with lower control limit (LCL) = $S_0 - h_0 = h_0$ and upper control limit (UCL) = $S_0 + h_1 = h_1$. Values above the UCL indicate an outbreak, i.e., rejection of the hypothesis of p_0 in favor of the hypothesis of p_1, although contrary to traditional control charts values beneath the LCL here do not indicate a rate decrease but rather acceptance of p_0 over p_1.

For the moving average (MA) charts, the moving average for the ith isolate with a “window” of size w (varied in different test conditions), $Y_{w,i}$, is calculated as

Publisher: CDC; Journal: Emerging Infectious Diseases
Article Type: Research; Volume: 8; Issue: 12; Year: 2002; Article ID: 01-0514
DOI: 10.3201/eid0812.010514; TOC Head: Research

$$Y_{w,i} = \begin{cases} \dfrac{X_i + X_{i-1} + \ldots + X_{i-w+1}}{w} = \dfrac{\sum_{j=i-w+1}^{i} X_j}{w}, & \text{for } i \ge w \\ \dfrac{X_i + X_{i-1} + \ldots + X_1}{i} = \dfrac{\sum_{j=1}^{i} X_j}{i}, & \text{for } i < w \end{cases} \quad (4)$$

This result then is compared to estimated upper (UCL) and lower *k*-sigma control limits for the *i*th isolate, LCL_i and UCL_i, with the standard deviation of the *i*th moving average, $\sigma_{Y,w,i}$, estimated by using the conventional moving range (*MR*) control chart method for individual data that occur over time,

$$\hat{\sigma}_{Y,w,i} = \frac{\hat{\sigma}_{X,i}}{\sqrt{\min(i,w)}} = \frac{\overline{MR}_i / 1.128}{\sqrt{\min(i,w)}}, \quad (5)$$

$$\overline{MR}_i = \frac{\sum_{j=2}^{i} \left| X_j - X_{j-i} \right|}{i-1}, \quad (6)$$

$$U\hat{C}L_i = \hat{\mu}_i + k\hat{\sigma}_{w,i} = \overline{X}_i + k\frac{\overline{MR}_i / 1.128}{\sqrt{\min(i,w)}}, \text{ and} \quad (7)$$

$$L\hat{C}L_i = \hat{\mu}_i - k\hat{\sigma}_{w,i} = \overline{X}_i - k\frac{\overline{MR}_i / 1.128}{\sqrt{\min(i,w)}}, \quad (8)$$

all for $i \geq 2$, where *i* is the current total number of data points, X_i is the *i*th data value, *w* is the size of the moving average, and $\overline{X}_i$ is the average of all data up to and including the *i*th data value. An MA value that exceeds its corresponding UCL will trigger an outbreak alert.
